# Interferon-Gamma Release Assays Versus Tuberculin Skin Test for Active Tuberculosis Diagnosis: A Systematic Review and Diagnostic Meta-Analysis

**DOI:** 10.3390/diagnostics15182343

**Published:** 2025-09-16

**Authors:** Muhammad Abubaker Tobaiqi, Musleh Naser Alshamrani, Shyamkumar Sriram, Ahmad Bakur Mahmoud, Hammad Ali Fadlalmola, Muayad Albadrani

**Affiliations:** 1 Department of Family and Community Medicine and Medical Education, College of Medicine, Taibah University, Madinah 42353, Saudi Arabia; 2 Preventive Medicine Department, Prince Sultan Armed Forces Hospital in Madinah, Madinah 42375, Saudi Arabia; 3 Department of Rehabilitation and Health Services, College of Health and Public Service, University of North Texas, Denton, TX 76203, USA; 4 Department of Clinical Laboratory Sciences, College of Applied Medical Sciences, Taibah University, Madinah 42353, Saudi Arabia; 5 Health and Life Research Center, Taibah University, Madinah 42353, Saudi Arabia; 6 Department of Community Health Nursing, Nursing College, Taibah University, Madinah 42353, Saudi Arabia

**Keywords:** sensitivity, specificity, tuberculosis diagnosis, tuberculin skin test (TST), interferon-gamma release assays (IGRAs)

## Abstract

**Background**: The world health goal of eliminating tuberculosis (TB) is heavily hinged on timely and efficient diagnosis and treatment. The interferon-γ release assays (I.G.R.A.s) can diagnose *Mycobacterium tuberculosis* infection and offer an alternative to the centuries-old tuberculin skin test (T.S.T.). Yet there is disagreement over replacing the T.S.T. with I.G.R.A.s as a standard tool. **Objective**: We aim to assess the diagnostic ability of I.G.R.A.s compared with T.S.T. for detecting active TB cases. **Methods**: A systematic review identified relevant studies from four databases. In the diagnostic meta-analysis conducted with OpenMeta Analyst software, we calculated the sensitivity (SN) and specificity (SP) for active TB detection via I.G.R.A. and T.S.T. methods compared to TB culture. Results included pooled estimates for SN and SP with 95% confidence intervals (CI), stratified by age, immunity, I.G.R.A. type, and T.S.T. cut-off. **Results**: Our meta-analysis revealed that TB diagnosis using T.S.T. showed an SN of 72.4% and SP of 79.3%, while I.G.R.A. demonstrated higher accuracy with an SN of 78.9% and SP of 85.7%. Subgroup analysis by age indicated that I.G.R.A. consistently outperformed T.S.T. in both adult and pediatric populations. Among immunocompromised individuals, T.S.T. had low SN (23%) but high SP (91.2%), whereas I.G.R.A. had higher SN (65.6%) but lower SP (81.9%). Immunocompetent subjects showed that T.S.T. had SN of 72% and SP of 87.3%, while I.G.R.A. had higher SN (82.9%) and SP (89.1%). Evaluation by I.G.R.A. type revealed that T-SPOT.GIT demonstrated a higher SN but lower SP compared to QFT-GIT. Assessing T.S.T. cut-offs, SP was highest (88.8%) at ≥15 mm, while SN peaked (71.6%) at ≥5 mm. **Conclusions**: I.G.R.A. consistently showed higher diagnostic accuracy than T.S.T. across most studied subgroups, indicating its potential superiority in active TB diagnosis.

## 1. Introduction

Tuberculosis (TB), a preventable and typically curable disease, remains a significant global health concern. Despite advancements in medicine, it was the second leading cause of death from a single infectious agent in 2022, surpassed only by COVID-19, and resulted in nearly double the number of deaths compared to HIV/AIDS [1]. Over 10 million new cases of TB are reported annually, underscoring the urgent need for action. Ending the global TB epidemic by 2030 is a crucial goal adopted by all Member States of the United Nations and the World Health Organization, requiring immediate and concerted efforts to achieve this target [1].

Ending TB as a public-health problem requires early diagnosis and effective treatment of active cases. Although the direct detection of TB bacilli in sputum through microscopy, culture growth or molecular tests remains the gold standard of diagnosis, they do not rule out TB in every patient suspected to be infected. In the case of patients with negative acid-resistant bacillus sputum smear microscopy, diagnosis and treatment decisions become challenging. Often, further TB diagnosis and effective management of active infections require other approaches [1,2].

The tuberculin skin test (T.S.T.) has remained a keystone of the public-health strategy for detecting LTBI and active TB due to its low cost, ease of use and limited cross-reactivity. However, its use has been limited by a high likelihood of false-positive results arising from prior *Bacillus Calmette-Guerin* (BCG) vaccination or infection with *non-tuberculous mycobacteria* (NTM) pathogens, and patients routinely undergo further testing to rule out these disorders. Recently, interferon-γ release assays (I.G.R.A.s) have emerged as the newest generation of testing to screen for infection with M tuberculosis. Both the commercially available T-SPOT.TB test and QuantiFERON-TB Gold assays exploit the unique M tuberculosis proteins absent in BCG and most environmental mycobacteria to improve specificity, largely overcoming the limitations of the T.S.T. [3,4].

Several meta-analyses have shown significantly improved sensitivity and specificity of I.G.R.A.s over the T.S.T. for detection of TB infection, but the stability of these diagnostic procedures remained unsettled [5,6,7,8,9,10]. To address this, the present meta-analysis aims to compare the diagnostic performance of I.G.R.A.s and T.S.T. in detecting active TB, considering patient-specific characteristics and epidemiological factors, which have been overlooked in previous studies.

## 2. Methods

Our study adhered to the Preferred Reporting Items for Systematic Reviews and Meta-analyses (PRISMA) guidelines and followed the recommendations outlined in the Cochrane Handbook for Systematic Reviews of Interventions [11,12].

### 2.1. Literature Search

An extensive literature search was conducted across multiple databases, including Web of Science, Scopus, PubMed, and Cochrane, covering publications up to May 2024. Additionally, a manual search of reference lists and meta-analyses was performed to identify further relevant citations. The search strategy involved combining various terms related to tuberculosis diagnostics, interferon-gamma release assays, and tuberculin skin tests: (“QFT” OR “T-SPOT” OR “SPOT” OR “Interferon-gamma Release Test*” OR “Release Test*, Interferon-gamma” OR “Test*, Interferon-gamma Release” OR “Interferon-gamma Release Assay*” OR “Interferon gamma Release Assay*” OR “Assay*, Interferon-gamma Release” OR “Interferon gamma Release Assay” OR “Release Assay*, Interferon-gamma” OR “I.G.R.A.”) AND (“Tuberculin Test” OR “Test, Tuberculin” OR “Tests, Tuberculin” OR “Tuberculin Tests” OR “T.S.T.” OR “PPD-B” OR “PPD B” OR “PPD-L” OR “PPD L” OR “Purified Protein Derivative of Tuberculin” OR “PPD” OR “PPD-S” OR “PPD-S” OR “PPD-CG” OR “PPD CG” OR “PPD-F” OR “PPD F”) AND (“Tuberculosis” OR “Tuberculoses” OR “Kochs Disease” OR “Koch’s Disease” OR “Koch Disease” OR “Infection*, *Mycobacterium tuberculosis*” OR “*Mycobacterium tuberculosis* Infection*”).

### 2.2. Eligibility Criteria

Two independent reviewers screened references and assessed their eligibility criteria. Studies were included in the meta-analysis if they met the following criteria: (1) enrollment of patients with active TB infection confirmed by a positive culture, (2) utilization of both I.G.R.A. and T.S.T. for assessing active TB, with culture as the gold standard, and (3) provision of essential data allowing the calculation of true-positive, false-positive, true-negative, and false-negative values. Exclusion criteria encompassed basic research, non-English publications, inaccessible full texts, and unpublished data.

### 2.3. Data Collection

A standardized data extraction process was employed using an offline data extraction sheet to gather pertinent information from each included study systematically. The extracted data encompassed: the first author and publication year, study location, active patient numbers, control numbers, age categories, I.G.R.A. type, T.S.T. cut-off values, number of participants who had BCG vaccination, inclusion criteria, study conclusions, and primary outcomes.

### 2.4. Quality Assessment

The methodological quality of the included studies was systematically evaluated utilizing the QUADAS-2 instrument, which comprehensively assesses study validity across four primary domains: participant recruitment and selection, index test methodology, reference standard criteria, and study flow and timing [13]. This evaluation enabled the appraisal of both bias risk and applicability concerns within these domains.

### 2.5. Data Synthesis

The meta-analysis was performed using the OpenMeta Analyst software v0.24.1, an open-source tool, to synthesize data and explore sources of heterogeneity. Sensitivity (SN) and specificity (SP) for active TB detection were calculated for both the I.G.R.A. and T.S.T. methods in comparison to the gold standard (TB culture), with sensitivity defined as the ratio of true positives to the sum of true positives and false negatives. Specificity was determined by dividing true negatives by the cumulative total of true negatives and false positives. The meta-analysis provided pooled sensitivity and specificity estimates, each accompanied by 95% confidence intervals (CI). Our analysis incorporated stratification based on age, immunity status, type of I.G.R.A., and T.S.T. cut-off values. Statistical heterogeneity was evaluated with I-squared (I^2^) and chi-squared (*X*^2^) statistics; *X*^2^ *p* < 0.10 and I^2^ ≥ 50% indicated significant heterogeneity.

## 3. Results

### 3.1. Study Selection

**Figure 1:**
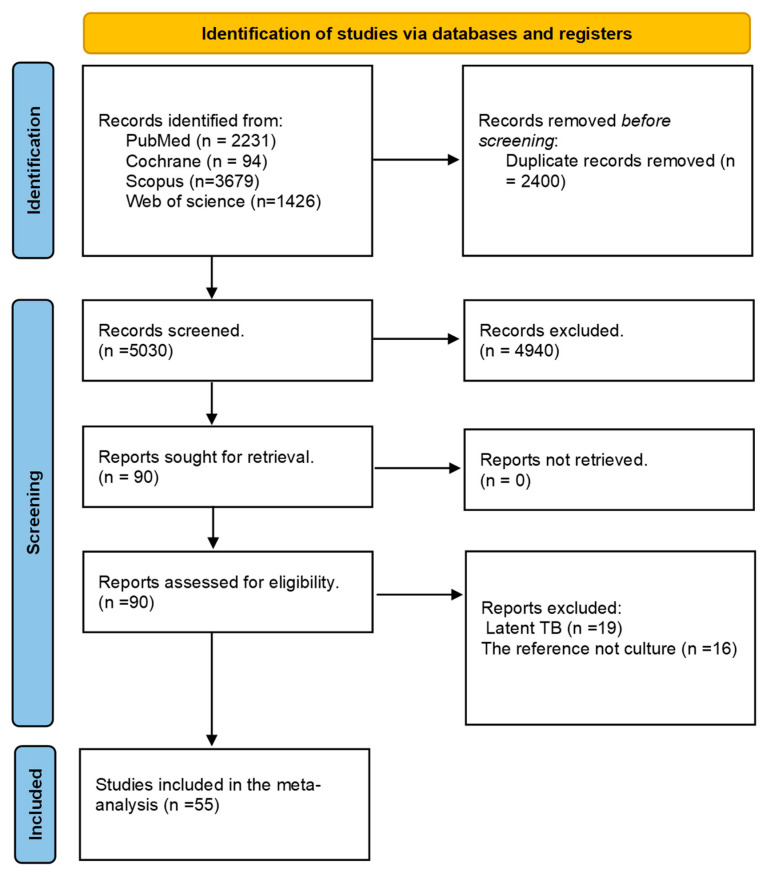
PRISMA flow chart.

An extensive literature search yielded an initial pool of 7430 studies, which, after removing duplicates, resulted in 5030 unique articles for further evaluation. Title and abstract screening narrowed the selection to 90 records for full-text screening. Of these, 35 studies were excluded based on predefined criteria. Eventually, 55 studies met the eligibility criteria and were incorporated into our systematic review and meta-analysis, ensuring a thorough and rigorous assessment of the available evidence [4,14,15,16,17,18,19,20,21,22,23,24,25,26,27,28,29,30,31,32,33,34,35,36,37,38,39,40,41,42,43,44,45,46,47,48,49,50,51,52,53,54,55,56,57,58,59,60,61,62,63,64,65,66,67]. The PRISMA flow diagram is shown in Figure 1.

### 3.2. Included Studies Characteristics

Our meta-analysis aggregated data from 55 studies, comprising a total of 3382 cases of active tuberculosis. The majority of the included studies were conducted in China and India. All studies evaluated the diagnostic performance of the T.S.T. and I.G.R.A.s for active tuberculosis, although the specific I.G.R.A. type utilized differed across studies, with the QuantiFERON-TB Gold In-Tube (QFT-GIT) assay being the most frequently employed. The study populations varied, with 24 studies exclusively enrolling adults, 30 studies focusing on pediatric populations, and one study [27], both adult and pediatric participants. A comprehensive summary of the included studies, including baseline characteristics. The TST cut-off values varied across studies (5 mm, 10 mm, or 15 mm). These thresholds were generally selected according to the epidemiological context: ≥5 mm was used in high-burden countries or immunocompromised patients to maximize sensitivity; ≥10 mm was commonly applied in intermediate-burden populations to balance sensitivity and specificity; and ≥15 mm was used in low-burden or BCG-vaccinated populations to reduce false positives. Several studies reported using multiple thresholds (5–15 mm) to allow comparison across settings as presented in Table 1.

### 3.3. Quality Assessment Results

**Figure 2:**
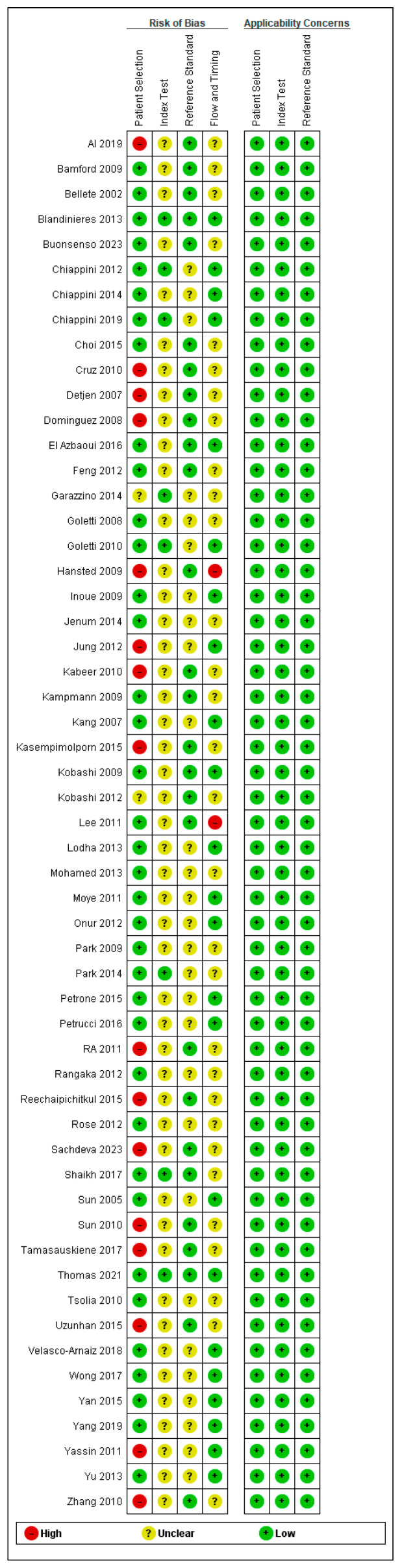
Quality assessment of the included studies.

Application of the QUADAS-2 tool revealed that the majority of included studies exhibited low applicability concerns across three domains: participant selection, index test methodology, and reference standard criteria. In contrast, assessment of bias risk indicated that most studies demonstrated a low risk of bias in the patient selection domain, whereas the index test domain was characterized by an unclear risk of bias. A detailed breakdown of these judgments is provided in Figure 2.

### 3.4. Diagnostic Meta-Analysis Outcomes

#### 3.4.1. Overall

**Figure 3:**
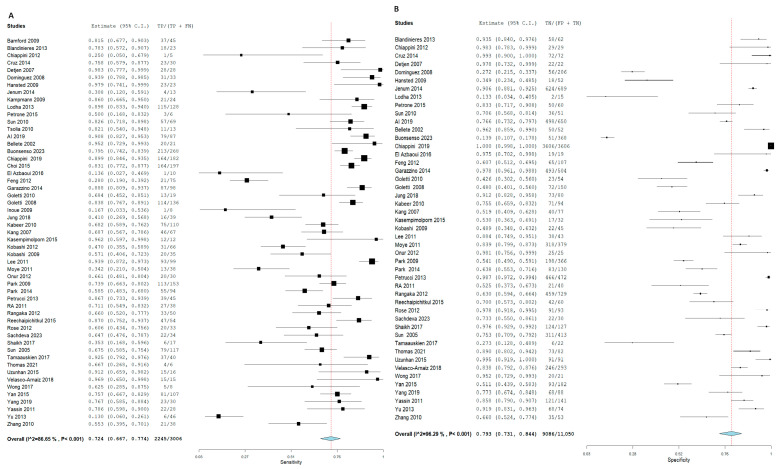
Forest plot of estimates of sensitivity and specificity for TB diagnosis via T.S.T in all population.

**Figure 4:**
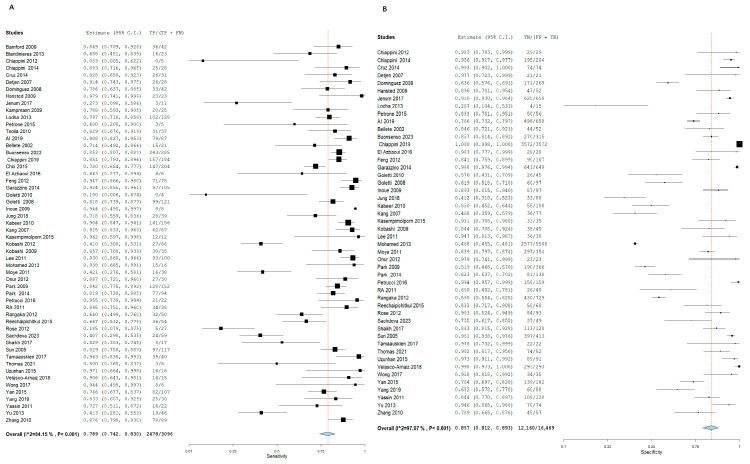
Forest plot of estimates of sensitivity and specificity for TB diagnosis via I.G.R.A in all population.

TB diagnosis via T.S.T. yielded an SN of 72.4% (95% CI: 66.7, 77.4) and an SP of 79.3% (95% CI: 73.1, 84.4). In contrast, I.G.R.A. demonstrated superior diagnostic accuracy, with an SN of 78.9% (95% CI: 74.2, 83) and an SP of 85.7% (95% CI: 81.2, 89.3). In the pooled studies for both approaches exhibited significant heterogeneity was observed across studies (I^2^ > 50%, *p* < 0.001). This heterogeneity likely reflects differences in geographic settings, the variety of IGRA platforms used, and the application of different TST cut-off values. Sensitivity analyses confirmed that our pooled estimates remained robust despite these variations.%. The high heterogeneity detected across studies highlights the influence of epidemiological setting, diagnostic platform, and threshold selection on test performance. While sensitivity analyses supported the stability of our findings, these variations must be considered when applying pooled estimates to local clinical practice, Figure 3 and Figure 4, respectively.

Forest plot of estimates of sensitivity and specificity: The red dotted line indicates the overall pooled estimate. The blue diamond represents the summary effect size with its 95% confidence interval. Each black square shows the effect estimate of an individual study, with the square size proportional to the study’s weight in the analysis. (A) Forest plot showing pooled sensitivity estimates with 95% confidence intervals for included studies. (B) Forest plot showing pooled specificity estimates with 95% confidence intervals for included studies. 

#### 3.4.2. According to Age

##### Adult

**Figure 5:**
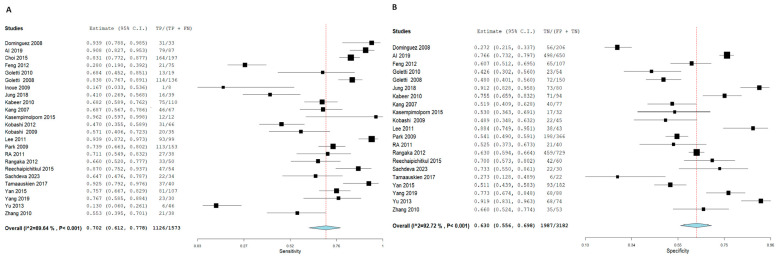
Forest plot of estimates of sensitivity and specificity for TB diagnosis via T.S.T in the adult population.

**Figure 6:**
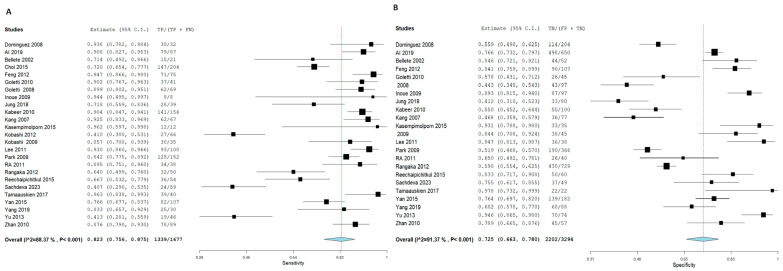
Forest plot of estimates of sensitivity and specificity for TB diagnosis via I.G.R.A in the adult population.

T.S.T. demonstrated an SN of 70.2% (95% CI: 61.2, 77.8) and an SP of 63% (95% CI: 55.6, 69.8) for active TB diagnosis. In contrast, I.G.R.A. exhibited superior diagnostic performance, with an SN of 82.3% (95% CI: 75.6, 87.5) and an SP of 72.5% (95% CI: 66.3, 78). Notably, significant heterogeneity was observed among the pooled studies for both approaches, characterized by χ^2^ *p* < 0.001 and I^2^ > 50%. Figure 5 and Figure 6, respectively.

Forest plot of estimates of sensitivity and specificity: The red dotted line indicates the overall pooled estimate. The blue diamond represents the summary effect size with its 95% confidence interval. Each black square shows the effect estimate of an individual study, with the square size proportional to the study’s weight in the analysis. (A) Forest plot showing pooled sensitivity estimates with 95% confidence intervals for included studies. (B) Forest plot showing pooled specificity estimates with 95% confidence intervals for included studies.

##### Children

**Figure 7:**
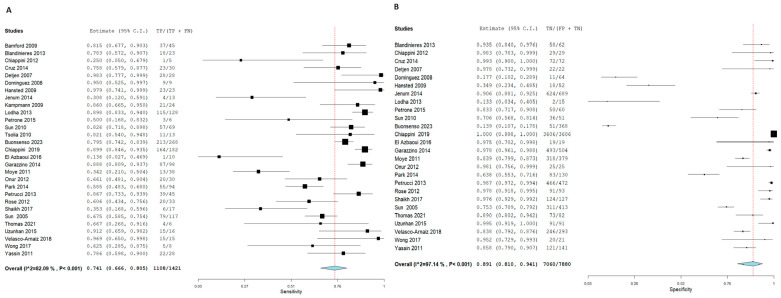
Forest plot of estimates of sensitivity and specificity for TB diagnosis via T.S.T in the children population.

**Figure 8:**
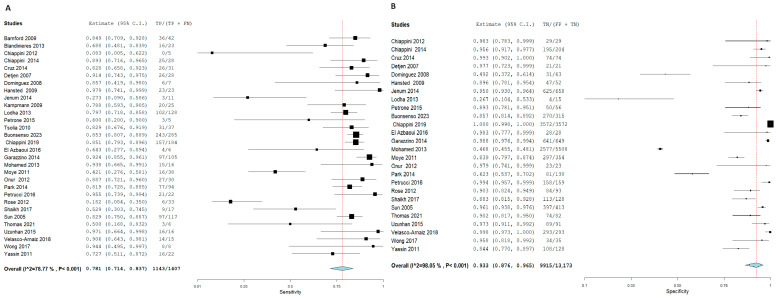
Forest plot of estimates of sensitivity and specificity for TB diagnosis via I.G.R.A in the Children population.

For T.S.T., the SN and SP for Active TB diagnosis were 74.1% and 89.1%, with corresponding 95% CI of [66.6, 80.5] and [81, 94.1], respectively. On the other hand, I.G.R.A. had a higher SN and SP as follows: 78.1% and 93.3% with corresponding 95% CI of [71.4, 83.7] and [87.6, 96.5], respectively. The pooled studies in both approaches were heterogeneous, with X^2^-*p* and I^2^ being < 0.001 and > 50%, respectively. Figure 7 and Figure 8, respectively.

Forest plot of estimates of sensitivity and specificity: The red dotted line indicates the overall pooled estimate. The blue diamond represents the summary effect size with its 95% confidence interval. Each black square shows the effect estimate of an individual study, with the square size proportional to the study’s weight in the analysis. (A) Forest plot showing pooled sensitivity estimates with 95% confidence intervals for included studies. (B) Forest plot showing pooled specificity estimates with 95% confidence intervals for included studies.

#### 3.4.3. According to the Immunity Status

##### Immunocompromised

T.S.T. yielded an SN of 23% (95% CI: 8.5, 48.8) and an SP of 91.2% (95% CI: 85.5, 94.8) for active TB diagnosis. In contrast, I.G.R.A. demonstrated a higher SN of 65.6% (95% CI: 34.5, 87.3) but a lower SP of 81.9% (95% CI: 36.9, 97.2). Notably, significant heterogeneity was observed among the pooled studies for both approaches, characterized by χ^2^ *p* < 0.001 and I^2^ > 50%, except for the SP of T.S.T., which exhibited homogeneity with χ^2^-*p* = 0.49 and I^2^ = 0%. Supplementary Materials Figures S1 and S2, respectively.

##### Immunocompetent

For T.S.T., the SN and SP for diagnosing active TB were 72% and 87.3%, with corresponding 95% CIs of [53.1, 85.4] and [73.5, 94.5], respectively. Conversely, I.G.R.A. exhibited higher SN and SP, at 82.9% and 89.1%, with corresponding 95% CIs of [76.3, 87.9] and [76.2, 95.4], respectively. The pooled studies for both methods were heterogeneous, with *X*^2^-*p* < 0.001 and I^2^ > 50%. Supplementary Materials Figures S3 and S4, respectively.

#### 3.4.4. According to I.G.R.A. Type

In the QFT-GIT, the SN and SP for diagnosing active TB were 78.8% and 85.6%, with corresponding 95% CIs of [73.4, 83.2] and [80.3, 89.6], respectively. On the contrary, T-SPOT.GIT demonstrated a higher SN but lower SP, at 80.6% and 83.7%, with corresponding 95% CIs of [72.5, 86.8] and [73.9, 90.3], respectively. The pooled studies for both approaches were heterogeneous, with *X*^2^-*p* < 0.001 and I^2^ > 50%. Supplementary Materials Figures S5 and S6, respectively.

#### 3.4.5. According to the T.S.T. Cut-Off Value

The diagnostic performance of T.S.T. at different induration cut-offs for active TB was evaluated. At ≥5 mm, SN and SP were 71.6% and 70.7%, respectively; at ≥10 mm, SN and SP were 70.6% and 75.8%, respectively; and at ≥15 mm, SN and SP were 62.7% and 88.8%, respectively. Heterogeneity was observed in pooled studies for all cut-offs. Collectively, the SP was highest in the subgroup with a cut-off value of ≥5 mm, reaching 88.8%. In contrast, the SN was highest in the ≥5 mm subgroup, at 71.6%. Supplementary Materials Figures S7–S9, respectively.

## 4. Discussion

Our diagnostic meta-analysis of 55 studies assessed active TB detection via T.S.T. and I.G.R.A. across various subgroups. Overall, I.G.R.A. showed superior accuracy over T.S.T., with higher SN and SP in most studied subgroups. Immunocompromised individuals showed varied results, with T.S.T. having higher SP but lower SN compared to I.G.R.A. Our subgroup analyses showed reduced diagnostic accuracy in immunocompromised, pediatric, and co-infected populations. In immunocompromised patients, impaired T-cell responses explain the lower sensitivity of both TST and IGRA. In children, immaturity of the immune system and higher rates of indeterminate IGRA results reduce reliability. In HIV/TB co-infected patients, immune dysregulation lowers concordance across tests. These findings highlight the importance of interpreting results within the context of patient characteristics. Reduced diagnostic accuracy in immunocompromised, pediatric, and co-infected populations can be attributed to biological and clinical factors. In immunocompromised patients, impaired T-cell responsiveness contributes to low sensitivity. In children, immature immune responses and a higher frequency of indeterminate IGRA results reduce test reliability. In HIV/TB co-infected populations, immune dysregulation limits the accuracy of both assays. These subgroup differences underscore the need for careful interpretation of diagnostic results based on patient characteristics. T-SPOT.GIT demonstrated a higher SN but lower SP compared to QFT-GIT. T.S.T.’s performance varied based on induration cut-offs, with a cut-off of ≥15 mm exhibiting the highest SP, while a cut-off of ≥5 mm had the highest SN. Our findings underscore the superior performance of I.G.R.A.s compared to the T.S.T. The heightened specificity of I.G.R.A.s translates to a reduction in false-positive results, thereby minimizing the need for additional, unnecessary tests and avoiding potential side effects from unwarranted treatments. Moreover, the enhanced sensitivity of I.G.R.A.s results in fewer false-negative outcomes, which is particularly crucial in the context of immunosuppressive therapy. Including I.G.R.A. in screening algorithms for individuals undergoing immunosuppressive treatment can potentially identify more TB infections.

The importance of early detection and intervention in tuberculosis control cannot be overstated, as it significantly contributes to successful patient outcomes and disease management [69,70,71]. However, we encounter a significant challenge with the conventional smear microscopy method for acid-fast bacilli, which often yields low detection rates, and the lengthy culture cycle required for *Mycobacterium tuberculosis* further impedes prompt diagnosis [69,70,71]. Consequently, the utility of microbiological techniques in this context is somewhat constrained.

T.S.T. is the recommended test for the diagnosis of tuberculosis because of the relative ease of performing it in non-laboratory settings with non-invasive procedures. In addition, it is less costly than I.G.R.A.s [72,73,74]. T.S.T. can be falsely positive in people vaccinated with the BCG vaccine or infected with *non-tuberculous mycobacteria*. It must be injected intradermally so that a consistent needle depth is inserted beneath the skin, and its interpretations are subjective, causing variability in results [72,73,74]. On the other hand, I.G.R.A.s require blood and specialized equipment. They are more expensive than T.S.T. and more difficult to give in low-income countries or in low-resource, non-laboratory settings. However, previous BCG vaccination or infection with *non-tuberculous mycobacteria* will not give false positive results with I.G.R.A.s. They also have less variability in results than T.S.T. [73,75,76,77]. However, I.G.R.A.s can be falsely indeterminate due to non-specifically high background reactivity or inadequate interferon-gamma response. If this happens, it is possible to repeat the test or to use another type of I.G.R.A. [73,75,76,77].

Our study revealed a notable variation in sensitivity and specificity across both the T.S.T. and I.G.R.A.s, which can be attributed to several factors, including epidemiological context, demographic differences, comorbidities, detection thresholds, and procedural variations. In order to minimize the heterogeneity of these factors and permit a more comparable assessment of T.S.T. versus I.G.R.A., we applied a strong selection criterion for both T.S.T. and I.G.R.A., if one was used, the other had to be as well within the same population, hence minimizing the number of studies deemed eligible. Second, by stratifying according to age groups, immunity status, type of I.G.R.A. and cut-off used for the T.S.T., we could compare the two diagnostic modalities more robustly. Significant heterogeneity was observed across studies (I^2^ > 50%, *p* < 0.001). This heterogeneity likely reflects differences in geographic settings, the variety of IGRA platforms used, and the application of different TST cut-off values. Sensitivity analyses confirmed that our pooled estimates remained robust despite these variations.

Our findings aligned with those of the UK Prognostic Evaluation of Diagnostic I.G.R.A.s Consortium (PREDICT) TB study conducted by Abubakar et al., which demonstrated the superior performance of I.G.R.A.s compared to the T.S.T. [78]. The UK PREDCTTB investigators found a greater difference in favor of the T-SPOT.TB assay. We similarly found that the T-SPOT.GIT had greater SN with a lower SP than QFT-GIT. One possible explanation for this difference could be the use of standardized cut-offs with the UK PREDCTB TB study, which could be the basis for the difference. In their 2011 study, Sester et al. compared the T.S.T. and I.G.R.A. methods by using active TB infection as a surrogate for latent TB infection (LTBI). They concluded that I.G.R.A.s demonstrated higher sensitivity compared to T.S.T. However, a direct comparison with our findings is challenging due to the differing cohorts utilized for T.S.T. and I.G.R.A. testing by Sester et al. [6]. Furthermore, a meta-analysis conducted by Diel et al. in 2010 supported the superior sensitivity of I.G.R.A.s over T.S.T. [69]. Nonetheless, direct comparability with our study is limited since only I.G.R.A.s were performed in the control population, precluding the determination of specificity [69].

Our findings, consistent with those of Dekeyser et al. and Nasiri et al., suggest that I.G.R.A.s are more sensitive and specific than T.S.T. in detecting TB infection [9,10]. In contrast, Auguste et al. (2017) found that the evidence was sparse and uncertain and did not indicate that I.G.R.A.s were superior to T.S.T. or vice versa [7]. The meta-analysis by Nasiri et al. reported pooled sensitivity and specificity values for T.S.T., QFT-G, and T-SPOT.TB, which was similar to our findings [10]. Similarly, Ai et al.’s study found that the interferon-γ release test was superior to T.S.T. as a screening tool for active tuberculosis [14]. However, our study differed from Auguste et al.’s (2019) study, which found no significant difference between I.G.R.A.s and T.S.T. in predicting progression to clinical tuberculosis [8].

This is the most extensive and updated meta-analysis comparing I.G.R.A. and T.S.T. for detecting active TB infections. Our study is distinguished by its comprehensive approach, incorporating stratifications based on age groups, immunity status, specific I.G.R.A. types, and T.S.T. cut-off points. Despite demonstrating higher accuracy, IGRA implementation faces practical challenges. Compared with TST, IGRA requires greater laboratory infrastructure, specialized equipment, and trained personnel. Costs are substantially higher, limiting feasibility in many high-burden, resource-limited settings. Policymakers should therefore evaluate cost-effectiveness and logistical feasibility before recommending IGRA as a replacement for TST. In such contexts, TST may remain the more practical option despite its lower accuracy. IGRAs require prompt sample processing and controlled laboratory conditions, which restrict their use outside urban or specialized centers. Reproducibility also varies by epidemiological context, with performance differences observed between high-prevalence and low-prevalence regions, and between rural and urban healthcare settings. These operational and contextual barriers limit the universal applicability of IGRA despite its superior accuracy. Furthermore, we applied stringent criteria by including only studies that utilized TB culture as the gold standard and those that compared I.G.R.A. and T.S.T. within the same study setting. These rigorous methods ensure the validity and consistency of our findings, offering valuable insights into the diagnostic efficacy of these tools in identifying active TB infections. While our study employed rigorous stratification methods, several limitations must be acknowledged. Future research should incorporate factors such as previous immunization and infection status by utilizing multivariate risk prediction models that account for prior TB exposure.

Emerging approaches such as machine learning may enhance TB diagnostics by integrating demographic, immunological, and epidemiological data. These predictive models could provide more nuanced interpretation of IGRA performance, particularly in populations with high BCG vaccination coverage or *non-tuberculous mycobacteria* exposure. This would enable a more comprehensive comparison between I.G.R.A.s and T.S.T. Additionally, the cost-effectiveness of T.S.T. and I.G.R.A. warrants consideration in upcoming studies. Other limitations include the retrospective nature of our study, potential selection bias introduced by physician discretion in choosing I.G.R.A. type and T.S.T. performance, and the lack of consideration for the endemic TB burden. To enhance the robustness of future studies, it is essential to account for these limitations and include stratification based on TB endemicity.

Future research should investigate the use of combined diagnostic strategies, such as integrating IGRAs with host biomarker panels or emerging molecular tools, to improve accuracy and provide rapid, point-of-care options. These combined approaches could enhance diagnostic precision and support global initiatives aimed at strengthening TB elimination strategies.

## 5. Conclusions

Our diagnostic meta-analysis reveals that I.G.R.A. outperforms T.S.T. in detecting active TB across different subgroups, but T.S.T. shows higher specificity in immunocompromised individuals. This suggests that patient characteristics should be considered when choosing a test. While our study used rigorous stratification methods, future research should address limitations such as prior TB exposure, immunization, and infection status, as well as cost-effectiveness comparisons between T.S.T. and I.G.R.A.

## Figures and Tables

**Table 1:** Summary and baseline characteristics of the included studies.

Study ID	Country	Recruitment Period	Number of Active Cases of TB	Number of Controls	BCG Vaccination, N (%)	Age Type	Age Range	Type of Interferon	TST Cut-Off Value	Conclusion
[14]	China	Between January and April 2016	30	650	-	Adult	1 to 96 years	IGRA	5 mm	“The interferon γ release test seems superior to TST and TBIgG as a screening tool for detecting active tuberculosis in China”.
[16]	United Kingdom	between January 2005 and December 2007	49	-	31 (63.3%)	Children	(2 months–16 years)	QFT-GIT T-SPOT.TB	15 mm	“A negative IGRA does not exclude active TB disease, but a combination of TST and IGRA enhances the sensitivity for identifying children with active TB”.
[17]	USA and Ethiopia	-	21	52	29 (16.6%)	-	Not mentioned	QFT-GIT	15 mm	“In its current form, with purified protein derivative used as the stimulation antigen, the IGRA was found to perform poorly in comparison to the TST in diagnosing M. tuberculosis infection”.
[18]	France	Between November 2007 to December 2011	51	31	46 (56%)	Children	0–15 years	QFT-GIT	10 mm	“In our low burden country, (i) QF-TB-IT specificity was 100%, (ii) QF-TB-It sensitivity was low in infants but commensurable to adult values in older children, and (iii) indeterminate results mostly relied on ongoing infections unrelated to TB”.
[20]	Spain	Between May 2017 and December 2019	488	-	235 (23.5%)	Children	less than 18 years	QFT-plus QFT-GIT T-SPOT.TB	5–10 mm	“The results indicate that the latest generation IGRA assay, QFT- Plus, does not perform better than previous generation IGRAs or the TST in children with TB disease. Overall, tests performed worse in CNS and miliary TB and in immunocompromised children. None of the tests evaluated had sufficiently high sensitivity to be used as a rule-out test in children with suspected TB”.
[23]	France	Between January 2009 and April 2010	5	29	5 (14%)	Children	Not specified	QFT-GIT	5–10 mm	“Our data suggest that IL-2 based ELISPOT with AlaDH antigen may be of help in discriminating children with active from those with latent TB”.
[22]	Italy	Between January 2010, and June 2013	28	-	-	Children	Not specified	T-SPOT.TB QFT-GIT	-	“In conclusion, according to our results, IGRA sensitivity in children below 5 years of age is particularly low and inferior to TST sensitivity. Considering that this group of children is at high risk for severe disease, the replacement of TST with IGRAs in young children appears to be unsafe. Our data suggest a high IGRA specificity in young children. Simultaneous use of TST and IGRA in BCG-vaccinated young children may be beneficial to avoid unnecessary treatment for LTBI”.
[21]	Italy	Between January 2010 to December 2017	205	3676	1954 (50%)	Children	0–18 years	QFT-GIT	-	“Our data suggest that QFT-IT might be used as a unique assay in children over 2 years of age investigated for recent immigration/adoption screening in cases of low-risk TB contact”.
[24]	USA	Between January 2005 and March 2012	204	-	-	Adult	Not specified	QFT-GIT	5–10 mm	“In San Francisco, QFT sensitivity was lower than that of TST, especially in patients with DM. Stratified analysis by sputum smear results demonstrated that this association was specific to smear-negative TB. In contrast, TST was not affected by the presence of DM”.
[25]	USA	Between 2005 and 2006	31	74	22 (20%)	Children	1 month to 18 years	T-SPOT.TB	-	“T-SPOT.TB is comparable to the TST in the diagnosis of tuberculosis disease and identification of high-risk children with tuberculosis infection and is more specific than the TST in children who have received the BCG vaccine”.
[26]	Germany	Between December 2004 to March 2006	28	22	4 (80%)	Children	4 months to 15 years	QFT-GIT	5 mm	“Both IGRAs demonstrated high diagnostic value in bacteriologically confirmed childhood TB. Their advantage in this study, when performed in addition to the TST, was the ability to distinguish-positive TST results caused by *non-tuberculous mycobacterial* disease, thereby reducing overdiagnosis of TB and guiding clinical management”.
[27]	Spain	Between September 2004 and November 2006	42	270	138 (44%)	Adult and children	0–18 years than 18 years	QFT-GIT T-SPOT.TB	5 mm	“Both gamma interferon tests were unaffected by prior Mycobacterium bovis BCG vaccination. Among children who were not BCG vaccinated but had a positive tuberculin skin test, QFN-G-IT was negative in 53.3% of cases, and T-SPOT.TB was negative in 50% of cases”.
[15]	Morocco	Between April 2011 to March 2015	10	28	-	Children	0 to 17 years	QFT-GIT	10 mm	“In epidemiological settings such as those found in Morocco, QFT-GIT is more sensitive than the TST for active TB diagnosis in children. Combining the TST and QFT-GIT would be beneficial for the diagnosis of active TB in children, in combination with clinical, radiological, and laboratory data”.
[28]	China	Between September 2008 and September 2009	75	107	182 (100%)	Adult	Not specified	T-SPOT.TB	10 mm	“IGRA could function as a powerful immunodiagnostic test to explore pulmonary and extrapulmonary TB, while TST failed to play a liable or auxiliary role in identifying TB disease and infection in the BCG-vaccinated population”.
[29]	Italy	Between October 2005 and April 2012	108	681	218 (26.5%)	Children	0 to 24 months	QFT-GIT	5 mm	“QTF-IT demonstrated good sensitivity and specificity, and a low rate of indeterminate results in the first 2 years of life, supporting its use at this age. However, considering costs and the similar performance between QTF IT and TST, it is reasonable to suggest the latter as first-line testing in young children. The complementary use of TST and interferon-γ release assays may be considered in selected cases to improve the accuracy of testing”.
[31]	India	Between April 2007 and March 2008	41	45	-	Adult	Not specified	QFT-GIT T-SPOT.TB	-	“IFN-g (but not IP-10, MCP-2, and IL-2) response to RD1 selected peptides is associated with active TB with a higher specificity than QFT-IT and TST”.
[30]	Italy and Spain	Between November 2005 and March 2008	173	197	159 (42%)	Adult	Not specified	QFT-GIT	10 mm	“The assay based on RD1 selected peptides has similar accuracy for active tuberculosis compared with TST and commercial IGRAs”.
[32]	Lithuania	Between January 2005 and February 2007	23	52	75 (100%)	Children	1–17 years	T-SPOT.TB	10 mm	“The T-cell-based method is more objective than the TST for identifying latent TB infection in children who had been previously BCG vaccinated. This method could be beneficial in countries like Lithuania, where TB is high despite high coverage with BCG vaccination. It may also help to avoid unnecessary chemoprophylaxis when TST reactions are false positive”.
[33]	Japan	Between April 2006 and June 2008	8	131	-	Adult	Not specified	QFT-GIT	5 mm	“Our data suggest that the QFT test is a beneficial supplementary tool for the diagnosis of active TB, even in dialysis patients. Negative and indeterminate results on this test may be used to exclude the presence of active TB”.
[34]	India	-	13	692	-	Children	0 to more than 2 years	QFT-GIT	10 mm	“The sensitivities of the TST and QFT for clinical TB in children < 3 Years of age were equally poor in this population. Stunted children were more susceptible to *Mycobacterium tuberculosis* infection and more prone to indeterminate QFT results. TST was less reliable in children with wasting”.
[35]	South Korea	-	32	-	-	Adult	Not specified	QFT-GIT T-SPOT.TB	10 mm	“The IGRAs and TST had no value as a single test either to rule in or rule out active TB in immunocompromised patients in an intermediate burden”.
[58]	India	Between November 2007 and October 2008.	162	100	-	Adult	More than 18 years	QFT-GIT	10 mm	“QFT-IT and IP-10 were highly sensitive in detecting active cases. The combination with TST improved the sensitivity of QFT-IT and IP-10 significantly. Although the higher sensitivity of the combination of QFT-IT/IP-10 and TST may be beneficial inactive TB diagnosis, they are limited by their poor specificity due to the high prevalence of latent TB in our settings”.
[36]	United Kingdom	Between February 2006 and February 2008	25	-	-	Children	2- months to 16 years	QFT-GIT T-SPOT.TB	10–15 mm	“A negative interferon-c release assay should not dissuade pediatricians from diagnosing and treating presumed active tuberculosis. If used for diagnosis of latent tuberculosis infection, interferon-c release assays could significantly reduce the number of children receiving chemoprophylaxis. Very good concordance between both tests was found”.
[37]	South Korea	Between December 2004 to December 2005	58	-	-	Adult	16 to 81 years	QFT-GIT T-SPOT.TB	10 mm	“High NPVs of QFT-G and T SPOT.TB for the diagnosis of active TB suggests the supplementary role of these tests for the diagnostic exclusion of active TB. However, the low PPV limits their beneficialness in routine clinical practice in South Korea, where the prevalence of latent TB infection is considerable”.
[38]	Thailand	-	12	33	-	Adult	1 to 58 years	QFT-GIT	5 mm	“The strip test did not appear to be beneficial for diagnosis of active TB in comparison with the current diagnostic standard. The assay may be particularly significant in situations where TB is clinically difficult to diagnose, like LTBI. It could be a meaningful tool in terms of high specificity and simplicity for ruling pediatric TB in countries with high TB infection rates. Further studies are needed to determine whether strip tests can be improved in their sensitivity and should be implemented into routine clinical practice”.
[40]	Japan	Between January 2005 and December 2007	35	45	-	Adult	Not specified	QFT-GIT	5 mm	“The QFT-2G appears to be a reliable diagnostic test, and in the appropriate clinical context, QFT-2G may be more beneficial than the TST in supporting a diagnosis of E-TB. Studies are needed to evaluate its value also in situations of low clinical probability”.
[39]	Japan	Between 2009 and 2010.	66	-	-	Adult	Not specified	QFT-GIT T-SPOT.TB	5 mm	“There were no significant differences among the three IGRA tests in this study. However, because the three IGRA tests demonstrated a significantly higher positive response rate for patients with pulmonary TB and a lower positive response rate for patients with non-pulmonary TB than TST, the three IGRA tests seemed to be more beneficial than TST for the differentiation of patients with pulmonary TB”.
[41]	South Korea	Between May 2008 and September 2009	41	43	-	Adult	20 to 29 years	QFT-GIT	10 mm	“Both the TST and QFT-IT demonstrated high sensitivity and specificity in differentiating active TB from other diseases. The diagnostic accuracy of these two tests did not differ significantly when applied to this clinical population of young, immunocompetent adults in whom neonatal BCG vaccination was common, there was no history of previous TB, and in whom suspicion of TB was high”.
[42]	India	-	128	15	-	Children	2 years to 15 years	QFT-GIT	10 mm	“In high-burden countries, QFT-GIT is comparable to TST and offers no added advantage in the diagnosis of childhood intrathoracic TB”.
[43]	South Africa	Between 2005 and 2007	16	5508	-	Children	12 to 18 years	QFT-GIT	10 mm	“The screening tools evaluated in this study may not be practical for routine use owing to low positive predictive values but may be beneficial in TB vaccine clinical trials”.
[44]	South Africa	Between July 2007 and September 2008	38	345	383 (100%)	Children	9 to 34 months	QFT-GIT	10–15 mm	“While TST and QFT had excellent concordance in this population, both tests had much lower sensitivity for TB disease than has been reported for other age groups. Our results suggested equivalent performance of QFT and TST in the diagnosis of TB disease in young children in a high-burden setting”.
[45]	Turkey	Between March 2008 and April 2009	30	25	47 (85%)	Children	3 months to 14 years	QFT-GIT	10 mm	“Utilization of QFT-GIT in the diagnosis of LTBI reduces false-positive results and prevents unnecessary treatment with INH and its adverse effects”.
[47]	South Korea	Between July 2007 to June 2008	153	-	-	Adult	Not specified	QFT-GIT	10 mm	“Additionally, the QFT-IT test has limited beneficialness in differentiating active pulmonary TB from *non-tuberculous mycobacterial* lung disease in areas with a high prevalence of latent tuberculosis infection”.
[46]	South Korea	Between October 2007 and April 2013	64	130	-	Children	Less than 18 years	QFT-GIT	10 mm	“Failure to enhance diagnostic yields by combination with other diagnostic modalities suggests that additional enforcement with IGRA may be insufficient to exclude other diagnoses in sputum smear-negative PTB suspects and to screen active PTB in an environment with intermediate TB prevalence and a high BCG vaccination rate”.
[48]	Uganda	Between May 2011 to September 2012	7	33	-	Children	1 month to 16 years	QFT-GIT	10 mm	“IP-10 levels are higher in children with respiratory illness compared to controls, independent of “TB status” suggesting that the evaluation of this parameter can be used as an inflammatory marker more than a TB test”.
[49]	Italy	-	45	30	4 (5%)	Children	0 to 14 years	QFT-GIT	10 mm	“Despite the concern about the use of QFT-IT in children because of their immature immune system, our results suggest the preferential use of QFT-IT as a support tool for diagnosis and management of TB, even in infants”.
[50]	South Korea	Between August 2004 to September 2007	38	40	38 (48%)	Adult	Not specified	QFT-GIT	10 mm	“Our findings indicate that the TST and IGRAs could not discriminate between active TB and MAC disease or latent TB infection in a TB-endemic area”.
[51]	South Africa	Between November 2007 and September 2009	50	729	-	Adult	31 to 42 years	QFT-GIT	5–15 mm	“QFT-GIT does not improve the discriminatory ability of current TB screening clinical algorithms used to evaluate HIV-infected individuals for TB ahead of preventive therapy. Evaluation of new TB diagnostics for clinical relevance should follow a multivariable process that goes beyond test accuracy”.
[52]	Thailand	Between September 2012 and March 2014	54	60	97 (85%)	Adult	6 to 83 years	QFT-GIT	10 mm	“The TST should be used as a screening test based on its higher sensitivity, whereas the QFT should be used as a confirmatory test because of its higher specificity”.
[53]	Tanzania	-	33	93	115 (91%)	Children	Less than 15 years	QFT-GIT	10 mm	“QFT and TST demonstrated poor performance and a surprisingly low sensitivity in children. In contrast, the performance of Tanzanian Zan adults was good and comparable to that of high-income countries. Indeterminate results in children were associated with young age and enhanced mortality. Neither test can be recommended for diagnosing active TB in children with immature or impaired immunity in a high-burden setting”.
[54]	India	Between July 2014 to September 2021	59	49	-	Adult	Not specified	IGRA	10 mm	“In a tuberculosis endemic region, IGRA had poor diagnostic accuracy for differentiating ITB from CD, suggesting a limited value of IGRA in this setting”.
[55]	India	Between August 2010 to December 2013	17	128	131 (90%)	Children	less than 5 years	T-SPOT.TB	5 mm	“The TST and the standard and novel ELISpot assays performed poorly in diagnosing active TB among young children in India”.
[56]	China	Between March 2011 to June 2014	117	413	486 (91.7%)	Children	less than 5 years	T-SPOT.TB	5–15 mm	“The results of the current study indicate that T-SPOT.TB has good sensitivity and specificity, supporting its use among patients of this age. A combination of IGRA and TST would be beneficial additions to assist in the diagnosis of childhood TB”.
[57]	China	Between July 2006 to December 2009	74	51	97 (77%)	Children	Not specified	T-SPOT.TB	10 mm	“Although IFN-γ release assay had relatively high sensitivity and specificity, we also should consider the higher costs and complexity of this test. Therefore, TSPOT could be used as the complementary tool of TST in circumstances when a suspected patient with negative TST results, or to exclude a positive TST result caused by BCG vaccination”.
[59]	Lithuania	-	40	22	-	Adult	More than 18 years	T-SPOT.TB	10 mm	“The T.SPOT.TB demonstrated greater accuracy in diagnosing TB than TST did. Positive T spot TB result but not the TST was more common in patients with diagnosed TB”.
[60]	India	-	6	82	-	Children	1 to 15 years	QFT-GIT	10 mm	“The higher sensitivity of the cheaper and simpler TST supports its use for TB diagnosis in a normally nourished population of BCG-vaccinated children”.
[68]	Greece	Between January 2007 and December 2008	11	-	-	Children	less than 15 years	QFT-GIT	-	“It is concluded that QuantiFERON-TB Gold-InTube compares with the tuberculin skin test in the diagnosis of TB disease and latent tuberculosis infection in TB contacts among children and has enhanced specificity”.
[61]	Turkey	-	16	92	-	Children	5 months and 17.5 years	QFT-GIT	-	“Although positive QFT-GIT test result is very significant for TB, negative results will a negative IGRA result cannot rule out TB with certainty infection. TST and QFT-GIT are used together may provide more efficient results”.
[62]	Spain	Between January 2005 and July 2015	15	293	-	Children	Less than 5 years	QFT-GIT	-	“In young BCG-unvaccinated children with recent TB contact, a dual testing strategy using TST and QFT-GIT in parallel may not be necessary. However, TST+/QFT-GIT negative discordance is common, and it remains uncertain if this constellation indicates TB infection or not. In active TB, QFT-GIT assays do not perform better than TSTs”.
[63]	Taiwan	-	7	35	-	Children	Less than 18 years	QFT-GIT	10 mm	“QFG-IT assay was more sensitive for the diagnosis of TB disease than TST in an intermediate burden population with universal neonatal BCG vaccination. The enhanced recognition of BCG-induced osteitis in recent years has alerted physicians that BCG induced lesions should be suspected when TST is positive but QFG-IT is negative”.
[64]	China	Between December 2011 and September 2012	107	182	289 (100%)	Adult	Not specified	T-SPOT.TB	5 mm	“Therefore, the results indicated that the T-SPOT.TB assay is a promising diagnostic test for active PTB in a BCG-vaccinated population, and should replace the TST. As the administration of anti-TB treatment resulted in a lower sensitivity to the diagnostic test, the T-SPOT.TB assay may also be suitable for the assessment of treatment outcomes”.
[64]	China	Between October 2016 and 2017	30	88	88 (74%)	Adult	18 to 95 years	T-SPOT.TB	10 mm	“The T-SPOT.TB test had a higher sensitivity than the TST, but the difference was not statistically significant. Neither the TSPOT.TB test nor the TST was sufficiently accurate to detect active M. tuberculosis infection”.
[65]	Ethiopia	-	28	156	100 (54%)	Children	1 to 15 years	QFT-GIT	10 mm	“Our findings therefore demonstrate that both INFc and IP10 identify children with latent and active TB. IP10 is less affected by the presence of HIV co-infection than INFc and has the potential to enhance the sensitivity of the IGRAS when used in combination with INFc. INFc, IP10 and TST however are unable to differentiate between latent and active disease”.
[66]	China	Between October 2010 and July 2012	46	74	-	Adult	Not specified	T-SPOT.TB	5 mm	“T-SPOT.TB is superior in screening ATB in HIV-infected patients in China over traditional TST. Additional TST would help to confirm a positive T-SPOT.TB result. Both tests work better for patients with extrapulmonary conditions”.
[67]	China	Between December 2006 to May 2008	89	57	129 (88%)	Adult	Not specified	T-SPOT.TB	5–10 mm	“The IGRA is a most promising test for both active TB and latent TB infection (LTBI) diagnosis due to the improvement of its specificity and convenience, especially in the Mycobacterium bovis BCG-vaccinated population. Furthermore, the T-SPOT.TB assay using ESAT-6 and CFP-10 in ATB patients during anti-TB treatment could serve as a potential predictor of therapeutic efficacy”.

Abbreviations: TST = Tuberculin skin tests; TB = tuberculosis; IGRA = interferon gamma release assay; QFT-GIT = quantiferon TB gold in tube.

## Data Availability

Data is available from the corresponding author upon reasonable request.

## Supplementary Materials

The following supporting information can be downloaded at: https://www.mdpi.com/article/10.3390/diagnostics15182343/s1, Figure S1: Forest plot of estimates of sensitivity and specificity for TB diagnosis via T.S.T in the Immunocompromised population; Figure S2: Forest plot of estimates of sensitivity and specificity for TB diagnosis via I.G.R.A in the Immunocompromised population; Figure S3: Forest plot of estimates of sensitivity and specificity for TB diagnosis via T.S.T in the Immunocompetent population; Figure S4: Forest plot of estimates of sensitivity and specificity for TB diagnosis via I.G.R.A in the Immunocompetent population; Figure S5: Forest plot of estimates of sensitivity and specificity for TB diagnosis via QFT-GIT; Figure S6: Forest plot of estimates of sensitivity and specificity for TB diagnosis via T-SPOT.TB; Figure S7: Forest plot of estimates of sensitivity and specificity for TB diagnosis via T.S.T as the cut-off value is ≥5 mm; Figure S8: Forest plot of estimates of sensitivity and specificity for TB diagnosis via T.S.T as the cut-off value is ≥10 mm; Figure S9: Forest plot of estimates of sensitivity and specificity for TB diagnosis via T.S.T as the cut-off value is ≥15 mm.

## References

[1] WHO Global Tuberculosis Report 2023. https://www.who.int/teams/global-tuberculosis-programme/tb-reports.

[2] Dinnes J., Deeks J., Kunst H., Gibson A., Cummins E., Waugh N., Drobniewski F., Lalvani A. (2007). A systematic review of rapid diagnostic tests for the detection of tuberculosis infection. Health Technol. Assess..

[3] Pai M., Zwerling A., Menzies D. (2008). Systematic review: T-cell-based assays for the diagnosis of latent tuberculosis infection: An update. Ann. Intern. Med..

[4] Yang J., Kong W., Xv N., Huang X., Chen X. (2019). Correlation between the tuberculin skin test and T-SPOT.TB in patients with suspected tuberculosis infection: A pilot study. Exp. Ther. Med..

[5] Sun L., Xiao J., Miao Q., Feng W., Wu X., Yin Q., Jiao W., Shen C., Liu F., Shen D. (2011). Interferon gamma release assay in diagnosis of pediatric tuberculosis: A meta-analysis. FEMS Immunol. Med. Microbiol..

[6] Sester M., Sotgiu G., Lange C., Giehl C., Girardi E., Migliori G.B., Bossink A., Dheda K., Diel R., Dominguez J. (2010). Interferon-γ release assays for the diagnosis of active tuberculosis: A systematic review and meta-analysis. Eur. Respir. J..

[7] Auguste P., Tsertsvadze A., Pink J., Court R., McCarthy N., Sutcliffe P., Clarke A. (2017). Comparing interferon-gamma release assays with tuberculin skin test for identifying latent tuberculosis infection that progresses to active tuberculosis: Systematic review and meta-analysis. BMC Infect. Dis..

[8] Auguste P., Madan J., Tsertsvadze A., Court R., McCarthy N., Sutcliffe P., Taylor-Phillips S., Pink J., Clarke A. (2019). Identifying latent tuberculosis in high-risk populations: Systematic review and meta-analysis of test accuracy. Int. J. Tuberc. Lung Dis..

[9] De Keyser E., De Keyser F., De Baets F. (2014). Tuberculin skin test versus interferon-gamma release assays for the diagnosis of tuberculosis infection. Acta Clin. Belg..

[10] Nasiri M.J., Pormohammad A., Goudarzi H., Mardani M., Zamani S., Migliori G.B., Sotgiu G. (2019). Latent tuberculosis infection in transplant candidates: A systematic review and meta-analysis on TST and IGRA. Infection.

[11] Higgins J.P.T., Thomas J., Chandler J., Cumpston M., Li T., Page M.J., Welch V.A., Higgins J.P.T., Thomas J., Chandler J., Cumpston M., Li T., Page M.J., Welch V.A. (2019). Cochrane Handbook for Systematic Reviews of Interventions.

[12] Page M.J., McKenzie J.E., Bossuyt P.M., Boutron I., Hoffmann T.C., Mulrow C.D., Shamseer L., Tetzlaff J.M., Akl E.A., Brennan S.E. (2021). The PRISMA 2020 statement: An updated guideline for reporting systematic reviews. BMJ.

[13] Whiting P.F., Rutjes A.W.S., Westwood M.E., Mallett S., Deeks J.J., Reitsma J.B., Leeflang M.M.G., Sterne J.A.C., Bossuyt P.M.M., QUADAS-2 Group (2011). QUADAS-2: A revised tool for the quality assessment of diagnostic accuracy studies. Ann. Intern. Med..

[14] Ai L., Feng P., Chen D., Chen S., Xu H. (2019). Clinical value of interferon-γ release assay in the diagnosis of active tuberculosis. Exp. Ther. Med..

[15] Azbaoui S.E., Sabri A., Ouraini S., Hassani A., Asermouh A., Agadr A., Abilkassem R., Dini N., Kmari M., Akhaddar A. (2016). Utility of the QuantiFERON^®^-TB Gold In-Tube assay for the diagnosis of tuberculosis in Moroccan children. Int. J. Tuberc. Lung Dis..

[16] Bamford A.R.J., Crook A.M., Clark J.E., Nademi Z., Dixon G., Paton J.Y., Riddell A., Drobniewski F., Riordan A., Anderson S.T. (2010). Comparison of interferon- release assays and tuberculin skin test in predicting active tuberculosis (TB) in children in the UK: A paediatric TB network study. Arch. Dis. Child..

[17] Bellete B., Coberly J., Barnes G.L., Ko C., Chaisson R.E., Comstock G.W., Bishai W.R. (2002). Evaluation of a Whole-Blood Interferon-g Release Assay for the Detection of *Mycobacterium tuberculosis* Infection in 2 Study Populations. Clin. Infect. Dis..

[18] Blandinières A., De Lauzanne A., Guérin-El Khourouj V., Gourgouillon N., See H., Pédron B., Faye A., Sterkers G. (2013). QuantiFERON to diagnose infection by *Mycobacterium tuberculosis*: Performance in infants and older children. J. Infect..

[19] Boom J.A., Tate J.E., Sahni L.C., Rench M.A., Quaye O., Mijatovic-Rustempasic S., Patel M.M., Baker C.J., Parashar U.D. (2010). Sustained Protection from Pentavalent Rotavirus Vaccination During The Second Year of Life AT A Large, Urban United States Pediatric Hospital. Pediatr. Infect. Dis. J..

[20] Buonsenso D., Noguera-Julian A., Moroni R., Hernández-Bartolomé A., Fritschi N., Lancella L., Cursi L., Soler-Garcia A., Krüger R., Feiterna-Sperling C. (2023). Performance of QuantiFERON-TB Gold Plus assays in paediatric tuberculosis: A multicentre PTBNET study. Thorax.

[21] Chiappini E., Storelli F., Tersigni C., Venturini E., de Martino M., Galli L. (2019). QuantiFERON-TB Gold In-Tube test performance in a large pediatric population investigated for suspected tuberculosis infection. Paediatr. Respir. Rev..

[22] Chiappini E., Bonsignori F., Mazzantini R., Sollai S., Venturini E., Mangone G., Cortimiglia M., Olivito B., Azzari C., Galli L. (2014). Interferon-Gamma Release Assay Sensitivity in Children Younger Than 5 Years is Insufficient To Replace The Use of Tuberculin Skin Test In Western Countries. Pediatr. Infect. Dis. J..

[23] Chiappini E., Della Bella C., Bonsignori F., Sollai S., Amedei A., Galli L., Niccolai E., Del Prete G., Singh M., D’Elios M.M. (2012). Potential Role of M. tuberculosis Specific IFN-γ and IL-2 ELISPOT Assays in Discriminating Children with Active or Latent Tuberculosis. PLoS ONE.

[24] Choi J.C., Jarlsberg L.G., Grinsdale J.A., Osmond D.H., Higashi J., Hopewell P.C., Kato-Maeda M. (2015). Reduced sensitivity of the QuantiFERON^®^ test in diabetic patients with smear-negative tuberculosis. Int. J. Tuberc. Lung Dis..

[25] Cruz A.T., Geltemeyer A.M., Starke J.R., Flores J.A., Graviss E.A., Smith K.C. (2011). Comparing the Tuberculin Skin Test and T-SPOT.TB Blood Test in Children. Pediatrics.

[26] Detjen A.K., Keil T., Roll S., Hauer B., Mauch H., Wahn U., Magdorf K. (2007). Interferon- Release Assays Improve the Diagnosis of Tuberculosis and Nontuberculous Mycobacterial Disease in Children in a Country with a Low Incidence of Tuberculosis. Clin. Infect. Dis..

[27] Domınguez J., Ruiz-Manzano J., Souza-Galvao M.D., Latorre I., Mila C., Blanco S., Jiménez M.A., Prat C., Lacoma A., Altet N. (2008). Comparison of Two Commercially Available Gamma Interferon Blood Tests for Immunodiagnosis of Tuberculosis. Clin. Vaccine Immunol..

[28] Feng Y., Diao N., Shao L., Wu J., Zhang S., Jin J., Wang F., Weng X., Zhang Y., Zhang W. (2012). Interferon-Gamma Release Assay Performance in Pulmonary and Extrapulmonary Tuberculosis. PLoS ONE.

[29] Garazzino S., Galli L., Chiappini E., Pinon M., Bergamini B.M., Cazzato S., Dal Monte P., Dodi I., Lancella L., Esposito S. (2014). Performance of interferon-γ Release Assay for the Diagnosis of Active or Latent Tuberculosis in Children in the First 2 Years of Age: A Multicenter Study of the Italian Society of Pediatric Infectious Diseases. Pediatr. Infect. Dis. J..

[30] Goletti D., Raja A., Kabeer B.S.A., Rodrigues C., Sodha A., Butera O., Carrara S., Vernet G., Longuet C., Ippolito G. (2010). IFN-γ, but not IP-10, MCP-2 or IL-2 response to RD1 selected peptides associates to active tuberculosis. J. Infect..

[31] Goletti D., Stefania C., Butera O., Amicosante M., Ernst M., Sauzullo I., Vullo V., Cirillo D., Borroni E., Markova R. (2008). Accuracy of Immunodiagnostic Tests for Active Tuberculosis Using Single and Combined Results: A Multicenter TBNET-Study. PLoS ONE.

[32] Hansted E., Andriuskeviciene A., Sakalauskas R., Kevalas R., Sitkauskiene B. (2009). T-cell-based diagnosis of tuberculosis infection in children in Lithuania: A country of high incidence despite a high coverage with bacille Calmette-Guerin vaccination. BMC Pulm. Med..

[33] Inoue T., Nakamura T., Katsuma A., Masumoto S., Minami E., Katagiri D., Hoshino T., Shibata M., Tada M., Hinoshita F. (2009). The value of QuantiFERON^®^TB-Gold in the diagnosis of tuberculosis among dialysis patients. Nephrol. Dial. Transplant..

[34] Jenum S., Selvam S., Mahelai D., Jesuraj N., Cárdenas V., Kenneth J., Hesseling A.C., Doherty T.M., Vaz M., Grewal H.M.S. (2014). Influence of Age and Nutritional Status on the Performance of the Tuberculin Skin Test and QuantiFERON-TB Gold In-Tube in Young Children Evaluated for Tuberculosis in Southern India. Pediatr. Infect. Dis. J..

[35] Jung J.Y., Lim J.E., Lee H., Kim Y.M., Cho S.-N., Kim S.K., Chang J., Kang Y.A. (2012). Questionable role of interferon-γ assays for smear-negative pulmonary TB in immunocompromised patients. J. Infect..

[36] Kampmann B., Whittaker E., Williams A., Walters S., Gordon A., Martinez-Alier N., Williams B., Crook A.M., Hutton A.-M., Anderson S.T. (2009). Interferon-γ release assays do not identify more children with active tuberculosis than the tuberculin skin test. Eur. Respir. J..

[37] Kang Y.A., Lee H.W., Hwang S.S., Um S.-W., Han S.K., Shim Y.-S., Yim J.-J. (2007). Usefulness of Whole-Blood Interferon-γ Assay and Interferon-γ Enzyme-Linked Immunospot Assay in the Diagnosis of Active Pulmonary Tuberculosis. Chest.

[38] Kasempimolporn S. (2015). Performance of a Rapid Strip Test for the Serologic Diagnosis of Latent Tuberculosis in Children. J. Clin. Diagn. Res..

[39] Kobashi Y., Abe M., Mouri K., Obase Y., Miyashita N., Oka M. (2012). Usefulness of Tuberculin Skin Test and Three Interferon-Gamma Release Assays for the Differential Diagnosis of Pulmonary Tuberculosis. Intern. Med..

[40] Kobashi Y., Mouri K., Yagi S., Obase Y., Miyashita N., Oka M. (2009). Clinical utility of a T cell-based assay in the diagnosis of extrapulmonary tuberculosis. Respirology.

[41] Lee J.E., Kim H.-J., Lee S.W. (2011). The clinical utility of tuberculin skin test and interferon-γ release assay in the diagnosis of active tuberculosis among young adults: A prospective observational study. BMC Infect. Dis..

[42] Lodha R., Mukherjee A., Saini D., Saini S., Singh V., Singh S., Grewal H.M.S., Kabra S.K., Delhi Tb Study Group (2013). Role of the QuantiFERON^®^-TB Gold In-Tube test in the diagnosis of intrathoracic childhood tuberculosis. Int. J. Tuberc. Lung Dis..

[43] Mahomed H., Ehrlich R., Hawkridge T., Hatherill M., Geiter L., Kafaar F., Abrahams D.A., Mulenga H., Tameris M., Geldenhuys H. (2013). Screening for TB in high school adolescents in a high burden setting in South Africa. Tuberculosis.

[44] Moyo S., Isaacs F., Gelderbloem S., Verver S., Hawkridge A.J., Hatherill M., Tameris M., Geldenhuys H., Workman L., Pai M. (2011). Tuberculin skin test and QuantiFERON^®^ assay in young children investigated for tuberculosis in South Africa. Int. J. Tuberc. Lung Dis..

[45] Onur H., Hatipoğlu S., Arıca V., Hatipoğlu N., Arıca S.G. (2012). Comparison of Quantiferon Test with Tuberculin Skin Test for the Detection of Tuberculosis Infection in Children. Inflammation.

[46] Park H., Shin J.A., Kim H.J., Ahn C.M., Chang Y.S. (2014). Whole Blood Interferon-γ Release Assay Is Insufficient for the Diagnosis of Sputum Smear Negative Pulmonary Tuberculosis. Yonsei Med. J..

[47] Park S.Y., Jeon K., Um S.-W., Kwon O.J., Kang E.-S., Koh W.-J. (2009). Clinical utility of the QuantiFERON-TB Gold In-Tube test for the diagnosis of active pulmonary tuberculosis. Scand. J. Infect. Dis..

[48] Petrone L., Cannas A., Aloi F., Nsubuga M., Sserumkuma J., Nazziwa R.A., Jugheli L., Lukindo T., Girardi E., Reither K. (2015). Blood or Urine IP-10 Cannot Discriminate between Active Tuberculosis and Respiratory Diseases Different from Tuberculosis in Children. BioMed Res. Int..

[49] Petrucci R., Lombardi G., Corsini I., Reggiani M.L.B., Visciotti F., Bernardi F., Landini M.P., Cazzato S., Dal Monte P. (2017). Quantiferon-TB Gold In-Tube Improves Tuberculosis Diagnosis in Children. Pediatr. Infect. Dis. J..

[50] Ra S.W., Lyu J., Choi C.-M., Oh Y.-M., Lee S.-D., Kim W.S., Kim D.S., Shim T.S. (2011). Distinguishing tuberculosis from *Mycobacterium avium* complex disease using an interferon-gamma release assay. Int. J. Tuberc. Lung Dis..

[51] Rangaka M.X., Gideon H.P., Wilkinson K.A., Pai M., Mwansa-Kambafwile J., Maartens G., Glynn J.R., Boulle A., Fielding K., Goliath R. (2012). Interferon release does not add discriminatory value to smear-negative HIV-tuberculosis algorithms. Eur. Respir. J..

[52] Reechaipichitkul W., Pimrin W., Bourpoern J., Prompinij S., Faksri K. (2015). Evaluation of the QuantiFERON?-TB Gold In-Tube assay and tuberculin skin test for the diagnosis of *Mycobacterium tuberculosis* infection in northeastern Thailand. Asian Pac. J. Allergy Immunol..

[53] Rose M.V., Kimaro G., Nissen T.N., Kroidl I., Hoelscher M., Bygbjerg I.C., Mfinanga S.G., Ravn P. (2012). QuantiFERON^®^-TB Gold In-Tube Performance for Diagnosing Active Tuberculosis in Children and Adults in a High Burden Setting. PLoS ONE.

[54] Sachdeva K., Kumar P., Kante B., Vuyyuru S.K., Mohta S., Ranjan M.K., Singh M.K., Verma M., Makharia G., Kedia S. (2023). Interferon-gamma release assay has poor diagnostic accuracy in differentiating intestinal tuberculosis from Crohn’s disease in tuberculosis endemic areas. Intest. Res..

[55] Shaikh N., Gupte A., Dharmshale S., Pokkali S., Thakar M., Upadhye V.J., Ordonez A.A., Kinikar A., Gupte N., Mave V. (2017). Novel interferon-gamma assays for diagnosing tuberculosis in young children in India. Int. J. Tuberc. Lung Dis..

[56] Sun L., Tian J., Yin Q., Xiao J., Li J., Guo Y., Feng G., Peng X., Qi H., Xu F. (2015). Performance of the Interferon Gamma Release Assays in Tuberculosis Disease in Children Five Years Old or Less. PLoS ONE.

[57] Sun L., Yan H., Hu Y., Jiao W., Gu Y., Xiao J., Li H., Jiao A., Guo Y., Shen A.-d. (2010). IFN-γ release assay: A diagnostic assistance tool of tuberculin skin test in pediatric tuberculosis in China. Chin. Med. J..

[58] Syed Ahamed Kabeer B., Raman B., Thomas A., Perumal V., Raja A. (2010). Role of QuantiFERON-TB Gold, Interferon Gamma Inducible Protein-10 and Tuberculin Skin Test in Active Tuberculosis Diagnosis. PLoS ONE.

[59] Tamašauskienė L., Hansted E., Vitkauskienė A., Miliauskas S., Naudžiūnas A., Šitkauskienė B. (2017). Use of interferon-gamma release assay and tuberculin skin test in diagnosing tuberculosis in Lithuanian adults: A comparative analysis. Medicina.

[60] Thomas L., Verghese V.P., Chacko A., Michael J.S., Jeyaseelan V. (2022). Accuracy and agreement of the Tuberculin Skin Test (TST) and the QuantiFERON-TB Gold In-tube test (QFT) in the diagnosis of tuberculosis in Indian children. Indian J. Med. Microbiol..

[61] Uzunhan O., Törün S.H., Somer A., Salman N., Köksalan K. (2015). Comparison of tuberculin skin test and QuantiFERON^®^-TB Gold In-Tube for the diagnosis of childhood tuberculosis. Pediatr. Int..

[62] Velasco-Arnaiz E., Soriano-Arandes A., Latorre I., Altet N., Domínguez J., Fortuny C., Monsonís M., Tebruegge M., Noguera-Julian A. (2018). Performance of Tuberculin Skin Tests and Interferon-γ Release Assays in Children Younger Than 5 Years. Pediatr. Infect. Dis. J..

[63] Wong K.-S., Huang Y.-C., Hu H.-C., Huang Y.-C., Wen C.-H., Lin T.-Y. (2017). Diagnostic utility of QuantiFERON–TB Gold In-Tube test in pediatric tuberculosis disease in Taiwanese children. J. Microbiol. Immunol. Infect..

[64] Yan L., Xiao H., Han M., Zhang Q. (2015). Diagnostic value of T-SPOT.TB interferon-γ release assays for active tuberculosis. Exp. Ther. Med..

[65] Yassin M.A., Petrucci R., Garie K.T., Harper G., Arbide I., Aschalew M., Merid Y., Kebede Z., Bawazir A.A., Abuamer N.M. (2011). Can Interferon-Gamma or Interferon-Gamma-Induced-Protein-10 Differentiate Tuberculosis Infection and Disease in Children of High Endemic Areas?. PLoS ONE.

[66] Yu Y., Zhao X., Wang W., Wu H., Chen M., Hua W., Wang H., Wei T., Jiao Y., Sun G. (2013). Diagnostic Performance of Interferon-Gamma Releasing Assay in HIV-Infected Patients in China. PLoS ONE.

[67] Zhang S., Shao L., Mo L., Chen J., Wang F., Meng C., Zhong M., Qiu L., Wu M., Weng X. (2010). Evaluation of Gamma Interferon Release Assays Using *Mycobacterium tuberculosis* Antigens for Diagnosis of Latent and Active Tuberculosis in *Mycobacterium bovis* BCG-Vaccinated Populations. Clin. Vaccine Immunol..

[68] Tsolia M.N., Mavrikou M., Critselis E., Papadopoulos N.G., Makrinioti H., Spyridis N.P., Kafetzis D.A. (2010). Whole blood interferon-γ release assay is a useful tool for the diagnosis of tuberculosis infection particularly among Bacille Calmette Guèrin-vaccinated children. Pediatr. Infect. Dis. J..

[69] Diel R., Loddenkemper R., Nienhaus A. (2010). Evidence-based comparison of commercial interferon-γ release assays for detecting active TB: A metaanalysis. Chest.

[70] Galloway K.M., Parker R. (2018). Could an increase in vigilance for spinal tuberculosis at primary health care level, enable earlier diagnosis at district level in a tuberculosis endemic country?. Afr. J. Prim. Health Care Fam. Med..

[71] Laurenti P., Raponi M., de Waure C., Marino M., Ricciardi W., Damiani G. (2016). Performance of interferon-γ release assays in the diagnosis of confirmed active tuberculosis in immunocompetent children: A new systematic review and meta-analysis. BMC Infect. Dis..

[72] Geldenhuys H., Verver S., Surtie S., Hatherill M., van Leth F., Kafaar F., Tameris M., Kleynhans W., Luabeya K.K., Moyo S. (2010). The tuberculin skin test: A comparison of ruler and calliper readings. Int. J. Tuberc. Lung Dis..

[73] Pinto L.M., Grenier J., Schumacher S.G., Denkinger C.M., Steingart K.R., Pai M. (2012). Immunodiagnosis of tuberculosis: State of the art. Med. Princ. Pract..

[74] ATS/CDC (2000). Targeted tuberculin testing and treatment of latent tuberculosis infection. American Thoracic Society. MMWR Recomm. Rep. Morb..

[75] Mazurek G.H., Jereb J., Vernon A., LoBue P., Goldberg S., Castro K., IGRA Expert Committee, Centers for Disease Control and Prevention (CDC) (2010). Updated guidelines for using Interferon Gamma Release Assays to detect *Mycobacterium tuberculosis* infection—United States, 2010. MMWR Recomm. Rep..

[76] Oni T., Gideon H.P., Bangani N., Tsekela R., Seldon R., Wood K., Wilkinson K.A., Goliath R.T., Ottenhoff T.H.M., Wilkinson R.J. (2012). Risk factors associated with indeterminate gamma interferon responses in the assessment of latent tuberculosis infection in a high-incidence environment. Clin. Vaccine Immunol..

[77] Banfield S., Pascoe E., Thambiran A., Siafarikas A., Burgner D. (2012). Factors associated with the performance of a blood-based interferon-γ release assay in diagnosing tuberculosis. PLoS ONE.

[78] Abubakar I., Drobniewski F., Southern J., Sitch A.J., Jackson C., Lipman M., Deeks J.J., Griffiths C., Bothamley G., Lynn W. (2018). Prognostic value of interferon-γ release assays and tuberculin skin test in predicting the development of active tuberculosis (UK PREDICT TB): A prospective cohort study. Lancet Infect. Dis..

