# Supplementary figures and images for: Interferon-Gamma Release Assays Versus Tuberculin Skin Test for Active Tuberculosis Diagnosis: A Systematic Review and Diagnostic Meta-Analysis
Source: Diagnostics (Basel). 2025 Sep 16;15(18):2343. doi: 10.3390/diagnostics15182343 (PMC12468246; doi:10.3390/diagnostics15182343)

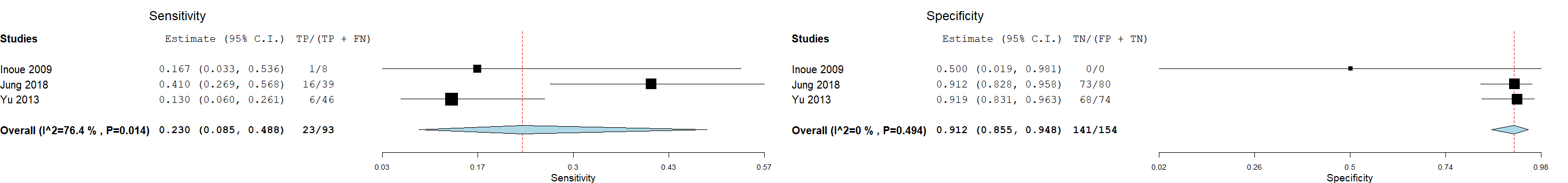

Supplement: Supplementary file 1 [file diagnostics-15-02343-s001.zip › Supplementary Figure S1.png]

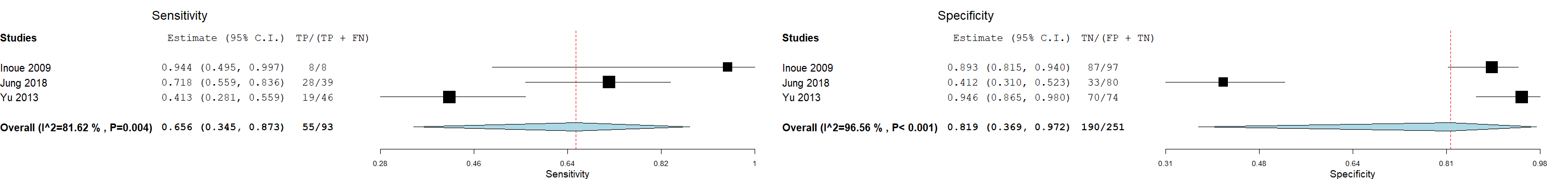

Supplement: Supplementary file 1 [file diagnostics-15-02343-s001.zip › Supplementary Figure S2.png]

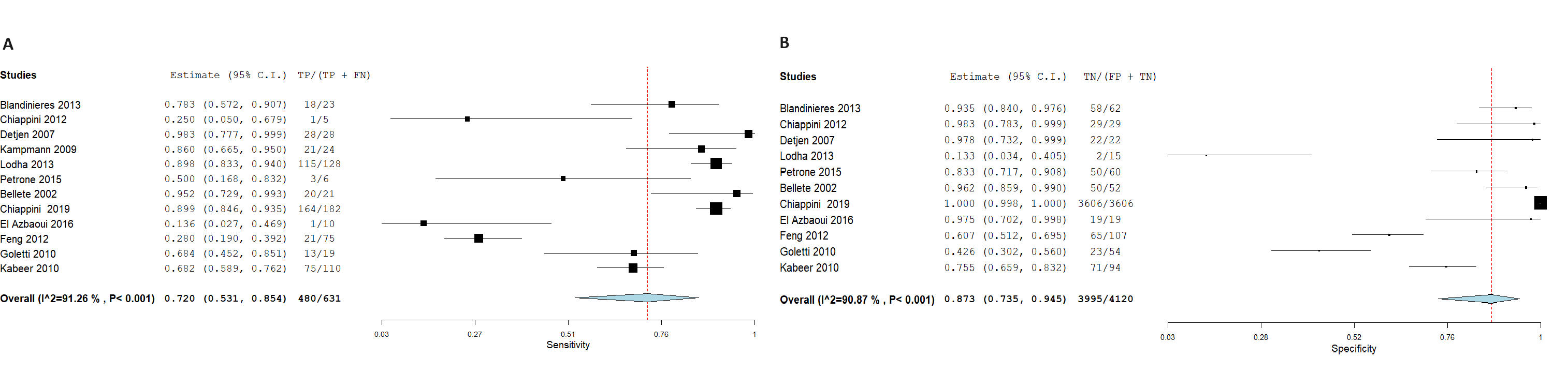

Supplement: Supplementary file 1 [file diagnostics-15-02343-s001.zip › Supplementary Figure S3.png]

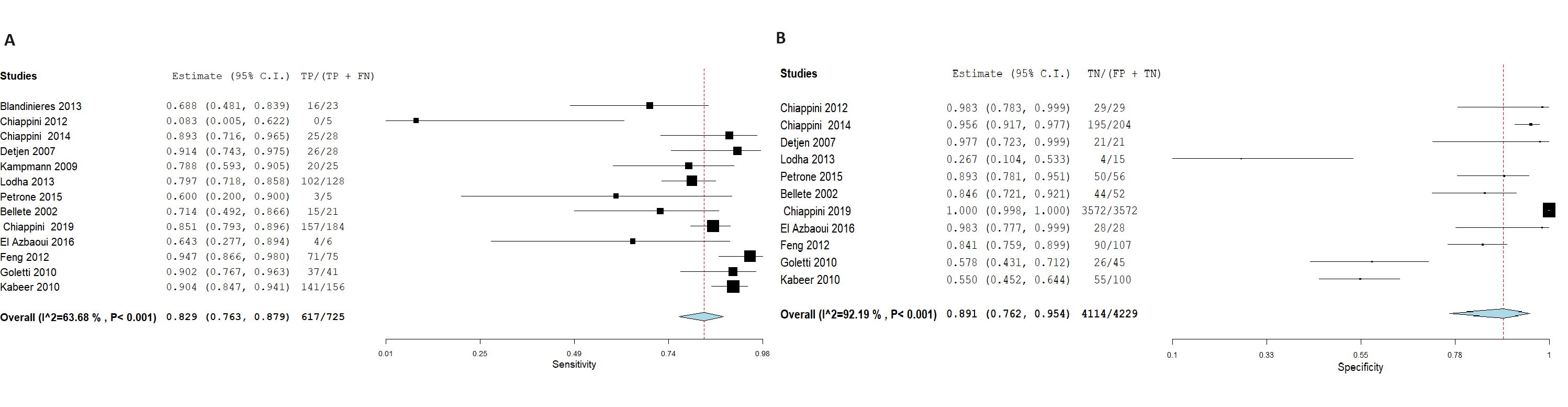

Supplement: Supplementary file 1 [file diagnostics-15-02343-s001.zip › Supplementary Figure S4.png]

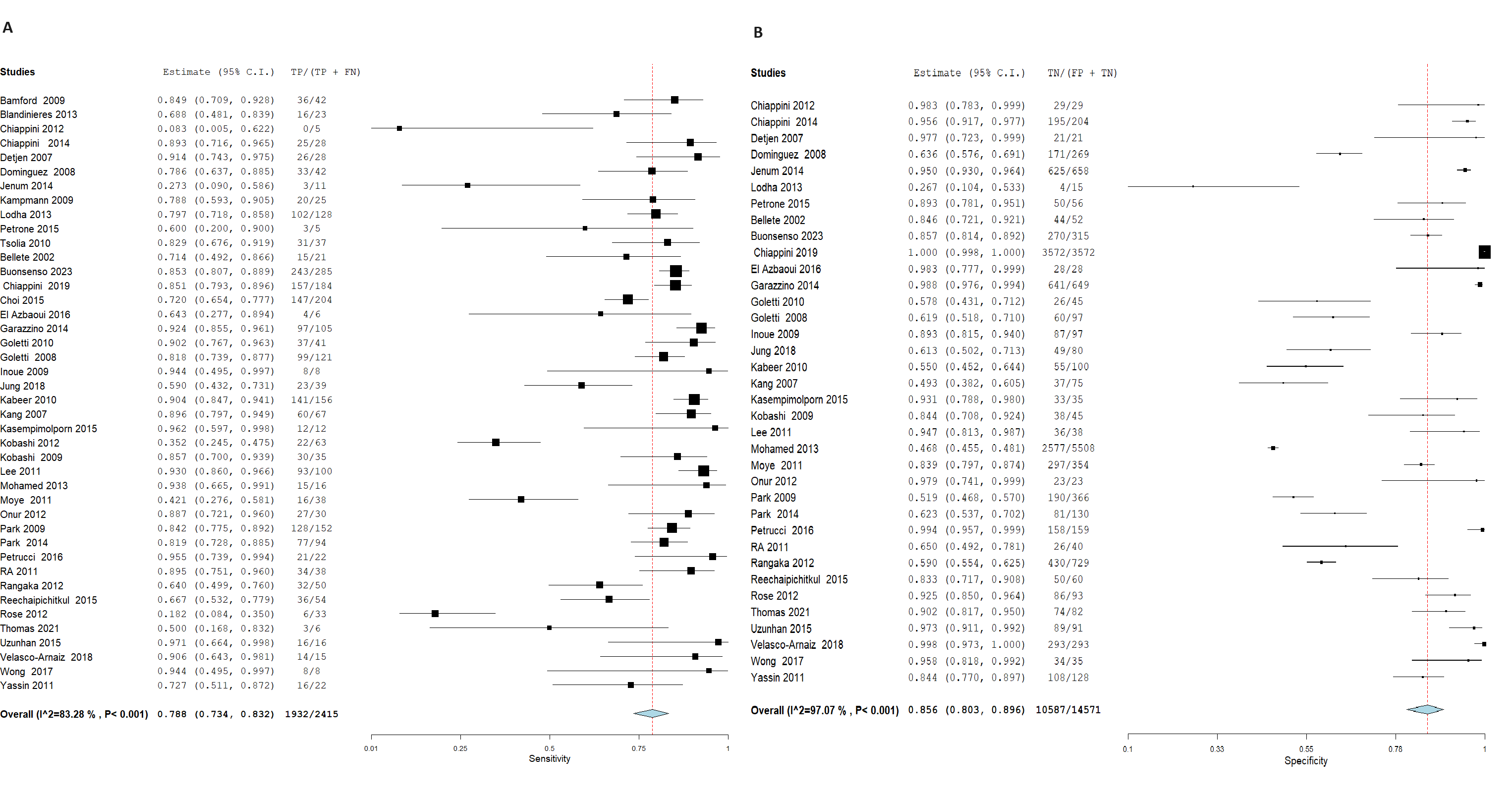

Supplement: Supplementary file 1 [file diagnostics-15-02343-s001.zip › Supplementary Figure S5.png]

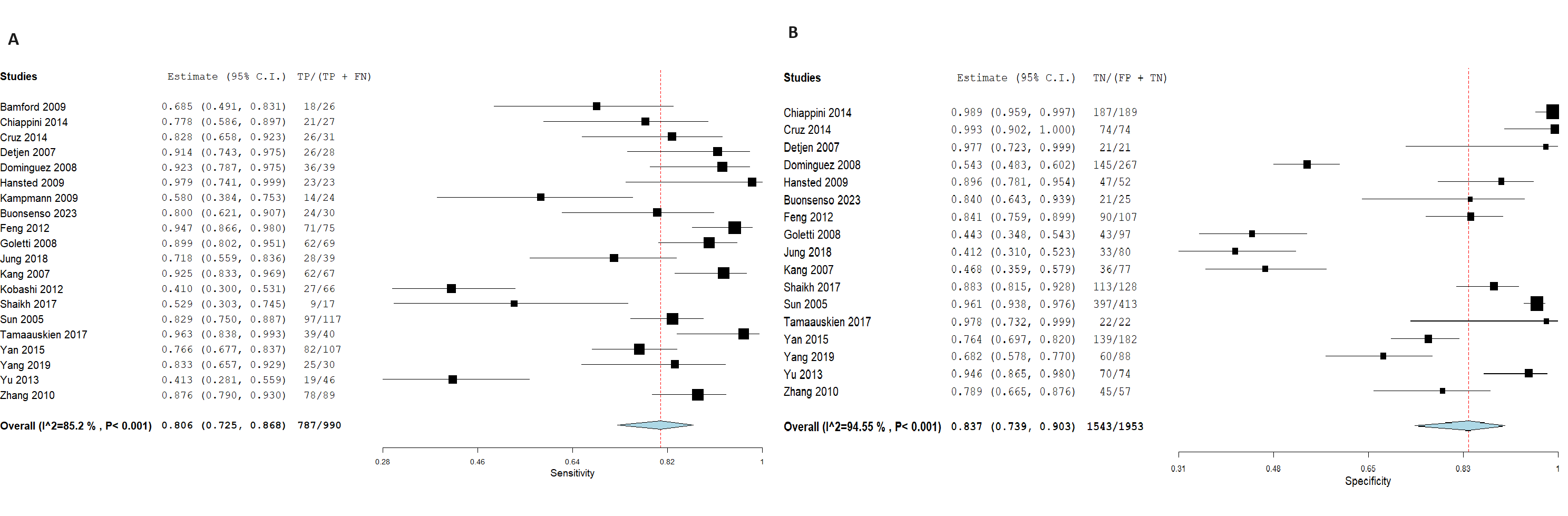

Supplement: Supplementary file 1 [file diagnostics-15-02343-s001.zip › Supplementary Figure S6.png]

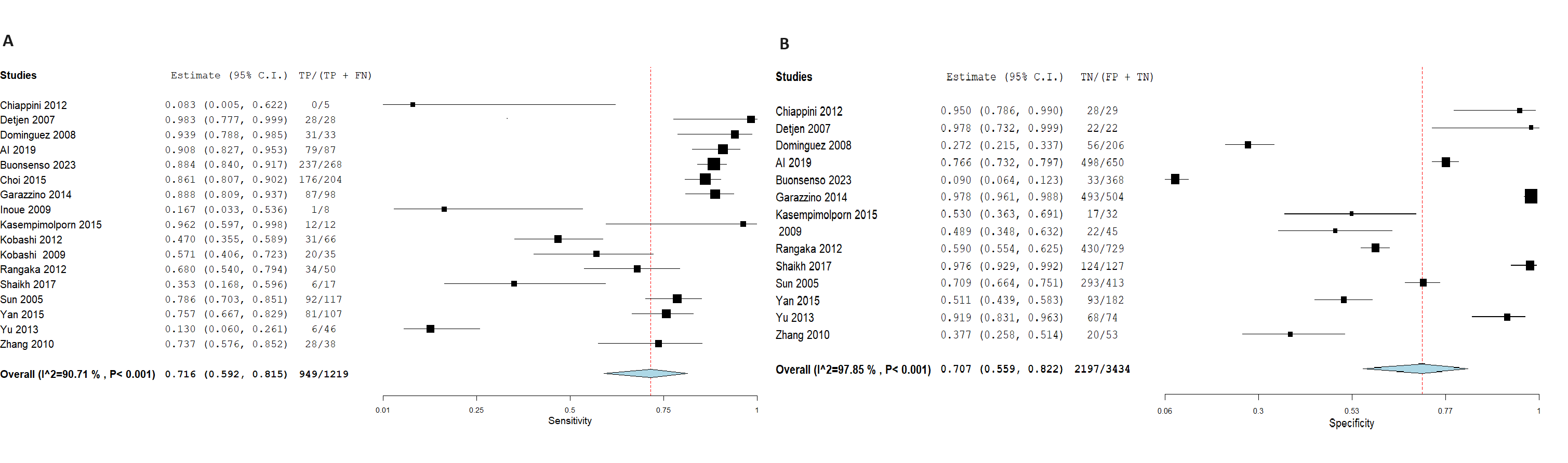

Supplement: Supplementary file 1 [file diagnostics-15-02343-s001.zip › Supplementary Figure S7.png]

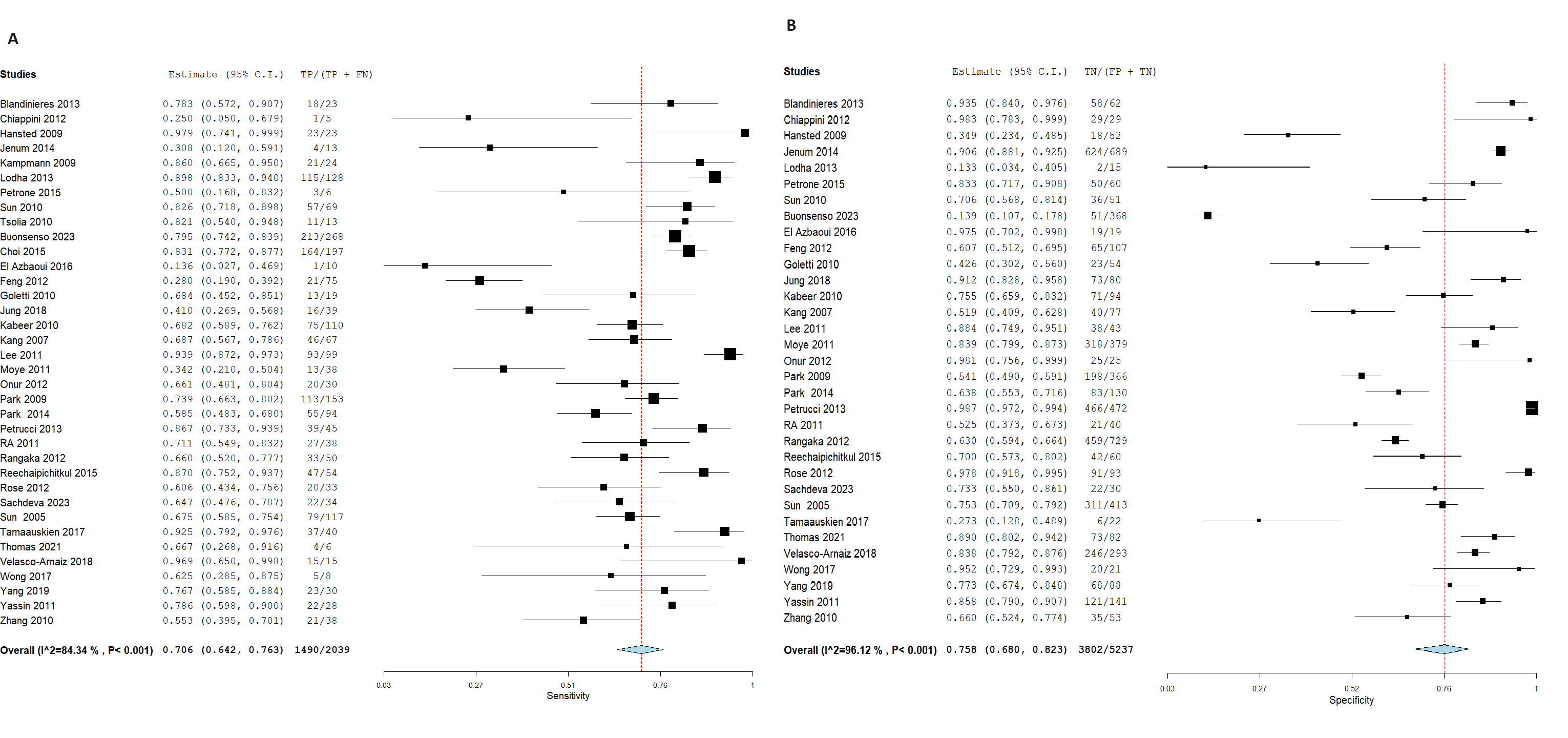

Supplement: Supplementary file 1 [file diagnostics-15-02343-s001.zip › Supplementary Figure S8.png]

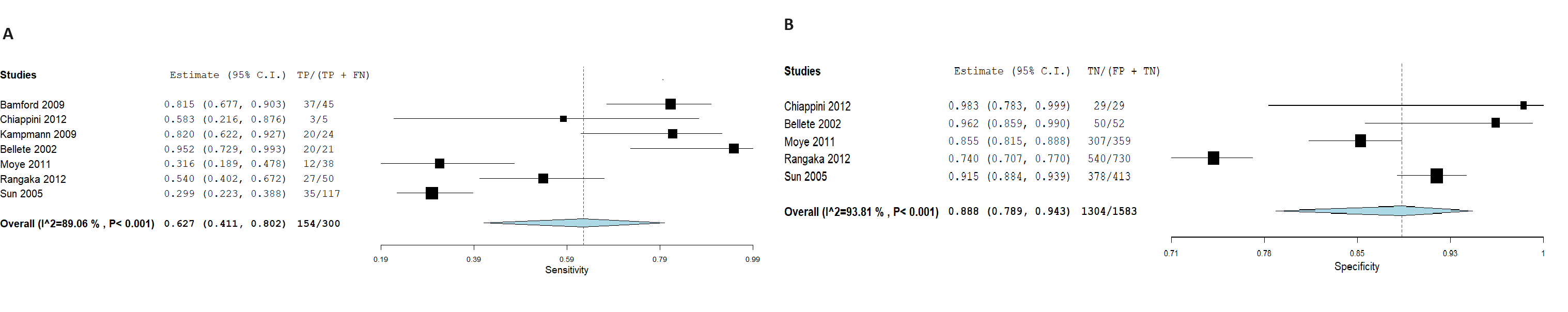

Supplement: Supplementary file 1 [file diagnostics-15-02343-s001.zip › Supplementary Figure S9.png]
